# Genome-Wide Analysis Revealed Homozygosity and Demographic History of Five Chinese Sheep Breeds Adapted to Different Environments

**DOI:** 10.3390/genes11121480

**Published:** 2020-12-09

**Authors:** Adam Abied, Lei Xu, Bahlibi W. Sahlu, Feng Xing, Abulgasim Ahbara, Yabin Pu, Jiang Lin, Haile Berihulay, Rabiul Islam, Xiaohong He, Joram M. Mwacharo, Qianjun Zhao, Yuehui Ma

**Affiliations:** 1 The Key Laboratory for Farm Animal Genetic Resources and Utilization, Ministry of Agriculture and Rural Affairs, Institute of Animal Science Chinese Academy of Agricultural Sciences (CAAS), Beijing 100193, China; aa.abied89@gmail.com (A.A.); puyabin@caas.cn (Y.P.); Jianglin@caas.cn (J.L.); haile.berihulay@yahoo.com (H.B.); md.rabiul27@yahoo.com (R.I.); hexiaohong@caas.cn (X.H.); mayuehui@caas.cn (Y.M.); 2 Dry Land Research Center (DLRC) and Animal Production, Agricultural Research Corporation (ARC), Khartoum 30, Sudan; 3 Institute of Animal Science (IAS), Chinese Academy of Agricultural Sciences (CAAS), Beijing 100193, China; xuleirock@163.com (L.X.); blenbah@gmail.com (B.W.S.); 4 College of Animal Science, Tarim University (TU), Alar 843300, Xinjiang, China; xingfeng2005@163.com; 5 School of Life Sciences, University of Nottingham, Nottingham NG7 2RD, UK; abulgasim68@gmail.com; 6 International Center for Agricultural Research in the Dry Areas (ICARDA), Addis Ababa 1108-2010, Ethiopia; j.mwacharo@cgiar.org

**Keywords:** high-density, SNPs, sheep, inbreeding, minor allele frequency

## Abstract

Homozygosity of long sequence genotypes are a result of parents transmitting identical haplotypes, which can be used to estimate their auto-zygosity. Therefore, we used high-density SNP Chip data to characterize the auto-zygosity of each breed according to the occurrence and distribution of runs of homozygosity (ROH). Subsequently, we identified the genomic regions with high runs of homozygosity frequencies within individuals of each breed. We selected 96 sheep samples from five local Chinese sheep breeds belonging to different geographical locations. We identified 3046 ROHs within the study breed individuals, among which the longer segments (>1–5 Mb) were dominant. On average, ROH segments covered about 12% of the genomes; the coverage rate of OAR20 was the lowest and that of OAR2 was the highest. The distribution analysis of runs of homozygosity showed that the detected ROH mainly distributed between >26 and 28 Mb. The Hetian and Hu sheep showed the lowest ROH distribution. The estimation of homozygosity level reflects the history of modern and ancient inbreeding, which may affect the genomes of Chinese indigenous sheep breeds and indicate that some animals have experienced recent self-pollination events (Yabuyi, Karakul and Wadi). In these sheep breeds, the genomic regions were assumed to be under selection signatures frequently in line with long ROH. These regions included candidate genes associated with disease resistance traits (*5S_rRNA*), the innate and adaptive immune response (*HERC2* and *CYFIP1*), digestion and metabolism (*CENPJ*), growth (SPP1), body size and developments (*GJB2* and *GJA3*). This study highlighted new insights into the ROH patterns and provides a basis for future breeding and conservation strategies of Chinese sheep breeds.

## 1. Introduction

The inheritance of consecutive homozygous fragments, identical haplotypes, of genome by the offspring from a common ancestor is known as runs of homozygosity (ROH) [1]. ROH is a useful indicator of genomic inbreeding and for identifying genomic regions that are potentially under historical selection pressure [2]. This is because the selection process may result in a high level of homozygosity, which is also known as homozygous operation [3,4]. Genetic diversity is a necessary raw material of evaluation and breeding and is the basis of natural and artificial selection [5]. However, to the best of our knowledge, the extent of ROH has not been widely investigated in the genome of different sheep breeds in China. This makes it crucial to assess and record levels of genetic variation within and between populations indicated by the inbreeding, heterozygosity, average minor allele frequency and single nucleotide ratio of polymorphisms. These indicators also provide information for breeding and conservation programs to effectively improve the production and reproductions levels, as well as to manage and conserve genetic resources [6]. Scanning the auto-zygosity may present inbred as well as non-bred populations due to several population phenomena, e.g. inbreeding, consanguineous matings, population bottleneck, genetic drift and natural and human-mediated selection [7,8]. Revealing the homozygosity of cattle genome, using high-density SNPs, has been an effective way to identify non-auto-zygosity identical by state (IBS) segments from auto-zygotic (IBD) [9]. Therefore, the description and identification of run of homozygosity may help in understanding the population structure, demographic history and evolution progress up to the present as well as unveiling footprints of natural or human-driven selection. In addition, the frequency and length of ROHs are both important parameters for determining causative forces for genomic changes over time. 

Effective population size (N_e_) is a population genetics basic concept. It determines the rate of evolutionary change caused by genetic drift and the equilibrium level of genetic variation and effective selection. N_e_ is often much lower than census size [10]. N_e_ is an important parameter for evaluation of population genetic diversity and can provide a powerful method to characterize and understand the genetic architecture underlying complex traits. It is very relevant to the conservation biology matrix because it is used to estimate of genetic drift rate and inbreeding rate as well as influence of systematic evolution such as selection, mutation, and migration and other demographic factors. Low N_e_ leads to inbreeding and reduces genetic variability [11]. 

Identification of long regions of homozygous genotypes in genome using high density SNP arrays has been considered an effective method for identifying the identity by descent (IBD) haplotypes [12,13]. In this regard, SNP arrays can provide information on past and more recent demographic changes, that is population size reflects founder effects and bottlenecks [3,14], which allow comparing the degree and pattern of homozygosity between populations with difference degrees of isolation and inbreeding [15,16] Different methods of inbreeding level estimation using genomic information were adopted. The importance of ROH in quantifying and understanding inbreeding in livestock, humans and plants was emphasized. The intense selection of livestock has alerted the scientific community to the need to protect the population [17], characterize and monitor auto-zygosity and maintain genetic diversity in long-term animal breeding programs [18,19]. Multiple studies have also shown that there is a relationship between ROH in the genome and the occurrence of recessive disorders (mainly, in humans) [13,20,21,22]. In recent years, the distribution and occurrence of ROH have been studied in cattle [4,23,24], pigs [3,25,26], goats [7] and sheep [27,28,29,30,31]. Population structure and selection could be evaluated according to the distribution and location of ROH. Inbreeding, population size reduction and selection could result in the long homozygous region in the genome [32,33]. In general, the similarity between the individuals at the whole genome level could be attributed to selection. Hence, ROH has an important role in recessive alleles mapping, which is related to the occurrence of diseases, as ROH has an increased risk of carrying IBD deleterious recessive alleles to disease occurrence. The risk of ROH carrying harmful recessive alleles of IBD increases, thus reducing the viability of organism [12,18,19]. However, there is limited information about genome selection and distribution of ROH throughout the genome and the effect of genome selection on inbreeding rate. Therefore, the purpose of this study was to characterize the run of homozygosity and identify ROH patterns as well as effective population sizes in the genomes of five local Chinese sheep breeds adapted to extremely dry and humid environment using high-density SNP chip data, which is essential for formulating new strategies and protecting genetic resources of sheep breeds. Our results provide insights into the patterns, evolutionary mechanisms and population demographics history of ROH.

## 2. Materials and Methods

All experiments carried out on animals in this study were fully approved by the Animal Protection and Use Committee (IAS-CAAS) of the Institute of Animal Science, Chinese Academy of Agricultural Sciences, Reference No: IASCAAS-AE-03, on 1 September 2014. 

### 2.1. Samples Collection and DNA Extraction

**Figure 1:**
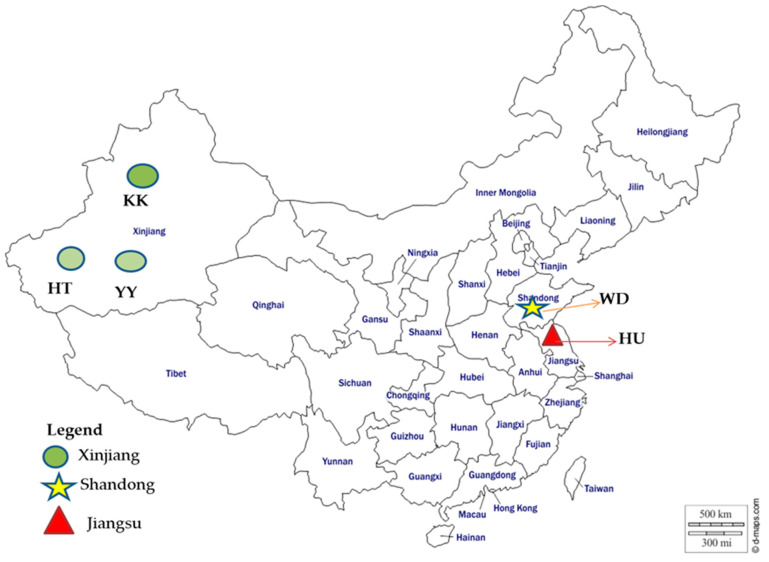
Map of China showing the different geographic distributions of the five Chinese indigenous sheep breeds analyzed in this study. The figure was taken from http://www.d-maps.com and adapted for illustrative purposes only.

In total, 96 Chinese sheep of five different local sheep breeds, four flocks for each breed, were selected and five individuals from each flock were sampled randomly. Ear tissue and/or whole blood samples were collected from Hetian, Karakul and Yabuyi sheep distributing in arid environment and Hu and Wadi sheep in humid environment (Figure 1 and Table 1). Ear marginal tissue samples were stored in 2 mL microcentrifuge tubes containing 75% ethanol. Genomic DNA was extracted from the ear tissue using a standard phenol–chloroform method [34]. Around 10 mL of blood were collected from the jugular vein of each animal into vacutainer tubes containing EDTA as anticoagulants and stored in ice/liquid nitrogen (−196 °C). DNA from the whole blood was extracted using DNeasy Blood and Tissue kit (Qiagen, Dusseldorf, Germany). The concentration and purity of DNA were measured by Nano-Drop 2000 spectrophotometer (Thermo Fisher Scientific Inc., Waltham, MA, USA) and stored at −20 °C for genotyping. 

### 2.2. Genotyping and Quality Control

DNA samples were genotyped using Ovine Infinium HD SNP Bead Chip (Beijing KPS Biotechnology CO Ltd., China), which contained 606,006 SNPs (600 K) of the Oar_v4.0 Ovine genome assembly. We used PLINK v1.9 software [35] for quality control (QC) of these SNPs. According to the procedures in [24], SNPs with call rate 95%, MAF < 0.05 and Hardy–Weinberg equilibrium (HWE) (*p* < 10^−5^) were filtered out. In addition, more than 10% of the samples missing genotyping (--geno) were deleted from the dataset. Finally, according to the suggestion of Zhang et al. [36] in PLINK manual, the indep-pairwise command parameters (SNP window size: 50; SNP moves per step: 5; *r*^2^ threshold: 0.2) were used to prune SNPs with high linkage disequilibrium (LD), which left 502,072 autosomal SNPs and 96 animals for further analysis. Pruning is necessary to produce a better comparison between populations, as some extended of SNPs have a lower minor allele frequency. LD decay patterns which can inform population demography were investigated for each sheep breed, with *r*^2^ values calculated with the parameter --*r*^2^--ld--window 1000--ld--window--*r*^2^ 0 commands in PLINK software.

### 2.3. Runs of Homozygosity Analysis

ROHs were identified and characterized in each of five sheep breeds using --homozyg function in the program PLINKv1.90, and the following conditions were adopted: minimum SNPs density were set to one SNP every 1 kB, and the maximum gap length was 1 Mb. For each ROH, one heterozygote and two missing genotypes were accepted. The total number of ROHs, the length of ROHs (in megabases) and the sum of all ROH segments (in megabases) were measured for all animals classified by breed and ROH length category. To compare the distribution of ROH among sheep breeds, we categorized the length of ROH into four classes (1–5, 5–10, 10–15 and >15 Mb). The average length of ROH was estimated by summing all ROH segments for each ROH length class per breed and divided by the total number of individuals of that respective breed. The genome inbreeding coefficients (F_ROH_) were calculated using the method previously described by McQuillan et al. [37]. The calculation method of F_ROH_ was as follows: F_ROH_ = (L_ROH_/L_AUT_), where L_ROH_ refers to the total length of ROH of each individual in the genome and L_AUT_ is the specific length of the autosomal genome covered by SNPs of sheep chips (2452.06 Mb). To verify the accuracy of (F_ROH_), we also used PLINK program and command --het to calculate the expected E(HOM) and observed O(HOM) number of homozygous genotypes and calculated the inbreeding coefficient (F_ROH_). 

### 2.4. Detection of Common Auto-Zygosity Islands

The --homozyg group functions implemented in PLINK v1.90 [35] was used to evaluate the shared ROH islands among individuals in order to identify the most common genome regions and candidate genes associated with ROH in five Chinese sheep breeds adapted to extremely humid and dry environments. The OAR_Rambouillet_V 1.0 model, detected on Ovine genome Ensembl (https://www.ensemble.org/Ovis_aries_rambuillet/info/index?db=core), was used for the identification of genes in ROH regions. In addition, the genome regions most commonly associated with ROH were identified by selecting the first top 0.5% (occurring in over 45% of the samples) of the common SNP observed in ROH [31,38]. Adjacent single nucleotide polymorphisms (SNPs) and incidence of ROH exceed the threshold used to form genomic regions called Island [36]. The percentage of SNPs present in ROH was calculated by counting the number of times the SNPs were detected in those ROH, which was plotted against the position of the SNP along the chromosomes. The candidate regions for highest ROH island identified were used for functional annotation of genes that were fully or partially continued within each selected region using DAVID v6.8 tools [39,40], and they were also used to identify significant (*p* ≤ 0.05). Gene Ontology (GO) terms and KEGG pathways used the list of genes from ROH islands and Ovine annotation file as backgrounds. Finally, we investigated the functional enrichments analysis for annotated genes in ROH Island from accurate literature and NCBI (https://www.ncbi.nlm.nih.gov/gene/) database.

### 2.5. Effective Population Size

The historical and modern effective population size (N_e_) for each of five sheep breeds was evaluated based on the following formula: *N*e = $\frac{1}{4c}\left(\frac{1}{E\left(r^{2}\right)}-1\right)$ [41], where N_e_ is effective population size, c is the genetic distance in Morgan and E (r^2^) is the expected r^2^ for distance c, implemented in the software SNeP v1.1 [42]. The N_e_ time points representing the number of generations ago (T) was calculated as *T* = $\frac{1}{2c}$ [43]. N_e_ was estimated for each chromosome and generation in the past.

## 3. Results

### 3.1. SNPs Filtration, Minor Alleles Frequency and Linkage Disequilibrium 

In this study, 606,006 SNPs were used before quality control; after filtering, the final number of animals and SNPs retained for analyses were 96 and 502,072, respectively, of the loci distributed over 27 autosomal chromosomes, which were used for downstream analysis. Zero SNPs were removed based on their results for Hardy–Weinberg Equilibrium (<10^−5^), minor allele frequency (<0.05) and call rate (<0.95). About 250,487 SNPs were removed using LD-based pruning when an *r*^2^ threshold of 5 was exceeded. After filtering, the 251,404 remaining SNPs were used for LD analysis. Minor allele frequencies were almost similar across the five breeds, inbreeding coefficients ranged between −0.004 in Wad sheep and −0.04 in Karakul sheep (Table 2). 

### 3.2. Runs of Homozygosity (ROH) Patterns

**Figure 2:**
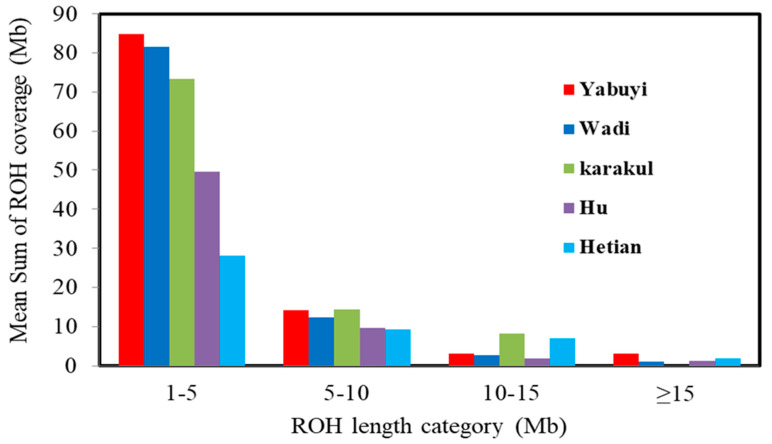
Genome-wide distribution of the mean sum of *F*(_ROH_) coverage different length categories per breed across five Chinese breeds. For each animal, within each ROH length category, ROH was summed up and then averaged per population.

The distribution of the relative number of ROH in different lengths between and within two sample groups in the five sheep breeds from extremely dry and humid environments is shown in Table 3. The average ROH category across the four classes was calculated; the lowest and highest length were observed on Hetain (11.56 Mb) and Yabuyi sheep (26.29 Mb), respectively (Table 3). In almost all breeds, most ROHs were >1–5 Mb class, followed by >5–10, 10–15 and ≥15. However, the proportion of major class groups (>1–5) varies between breeds: at least 80% of ROH length classes are in Yobuyi and Wadi sheep; 70% in Karakul breed; 50% in Hu sheep; and 28% in the Hetian sheep breed (Table 3 and Figure 2). In addition, Karakul and Yabuyi breeds had the highest frequencies in length classes (5–10 Mb). The higher levels of ROH classes (≥1–5) across breeds ranged from 23.84 Mb in Hetian to 90.17 Mb in Yobuyi breeds from dry environments, while the lowest levels of classes (≥15) ranged from zero in Karakul to 3.12 Mb in Yabuyi sheep from dry environments.

**Figure 3:**
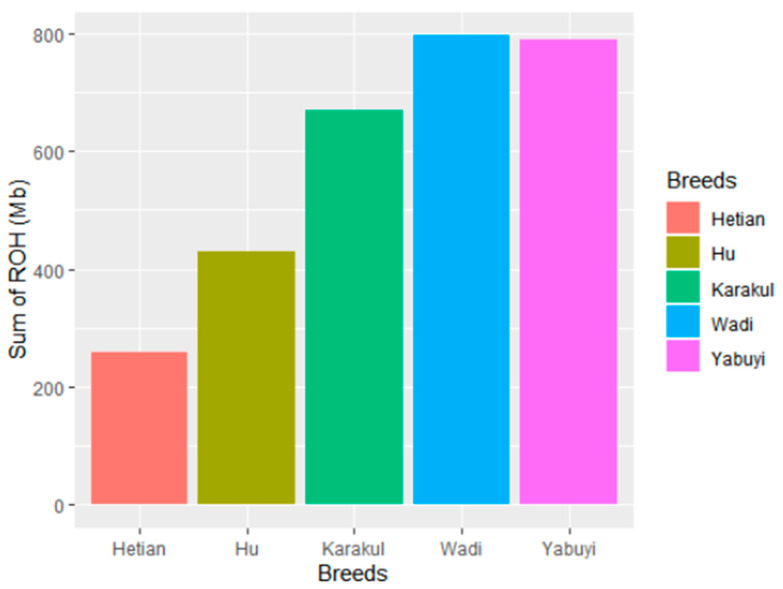
Runs of homozygosity patterns length of the genome across five Chinese sheep breeds.

According to the set parameters, 3046 ROHs were detected in five sheep breeds, with an average of 35.84 per individual. The genome length ranged between 273 and 984 Mb in five populations including 96 individuals who had at least one ROH. The number of ROH differed significantly between breeds. The Wadi sheep displayed the largest number of ROH (796), followed by Yabuyi (790) on average (means of 46.8 and 46.5 Mb respectively). The lowest number of ROH (259) was found in the Hetian sheep breed on average (mean of 15.23 Mb) (Figure 3).

**Figure 4:**
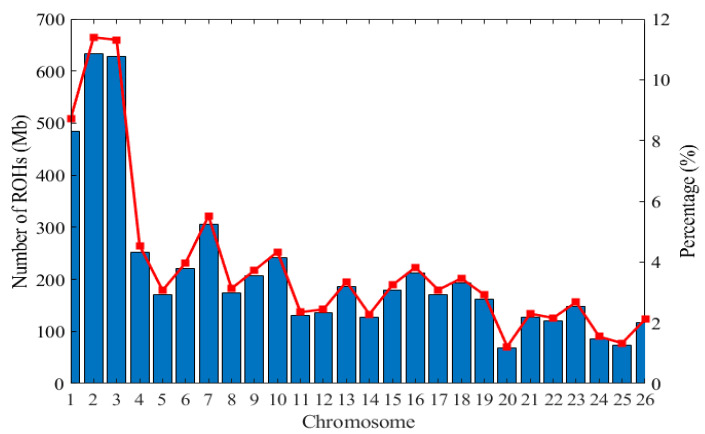
Distribution of the runs of homozygosity across the chromosome in 96 individuals. The X-axis denotes the number of ROHs longer than 1 Mb per chromosome and the Y-axis represents the average percentage (%) of each chromosome covered by ROH (red lines).

The average length of ROH was 2.27 Mb and the longest segment across breeds detected on chromosome OAR2 was 633.04 Mb, which was composed of 121,567 SNPs, followed by OAR3 with 628.49 Mb and 116,049 SNPs. The shortest length of ROH was detected in OAR20 (67.63 Mb) having 13,838 SNPs. In general, the total number of ROHs per chromosome reduces with decrease in chromosome length. The highest percentages of ROH per chromosome was observed on chromosome OAR2 (11.39%) and OAR3 (11.31%), whereas the lowest length was on chromosome OAR20 (1.23%) (Figure 4). The correlation between the number of ROH (n ROH) and the sum of all ROH segments was *R*^2^ = 0.683 (Figure S1). 

### 3.3. Genomic Regions with a High ROH Frequency

**Figure 5:**
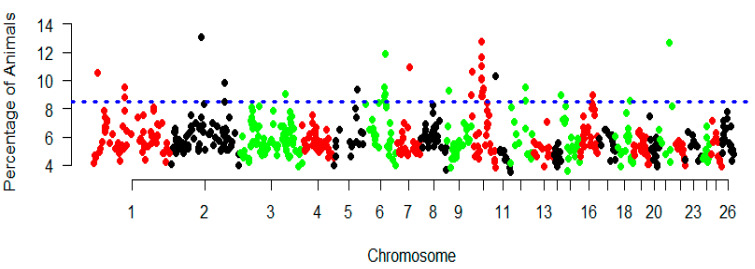
Manhattan plot of incidence of each SNP in the ROH across individuals. The dashed line represents the 45% threshold.

Auto-zygosity islands are obvious across the whole genome and their distributions varied in length and location on chromosomes (Table S1). The most common genomic regions associated with high frequency of ROHs occurrences were detected in five sheep breeds, and the percentage of SNPs was assessed by calculating the frequency of SNPs in these ROHs on the autosomal chromosomes, as shown in the Manhattan plot (Figure 5). To identify genomic regions that were most commonly associated with ROH in all individuals, the top 0.5% of SNPs [44] with the common highest occurrences (occurring in over 45% of the samples) in ROH were considered as candidate SNPs (Figure 5). In total, eight ROH island regions including 28 genes were identified across the 26 autosomes. The strongest candidate regions were identified on chromosomes OAR2, OAR10, OAR21 and OAR6 spanning several genes, e.g. *ANXA10*, *CRYL1*, *PAG4* and *5S_rRNA* genes, respectively (Table 4). The top three GO terms and KEGG pathways were connexin complex (GO: 0005922), aspartic-type endopeptidase activity (GO: 0004190) and protein digestion and absorption (oas04974) (Table S2).

### 3.4. Effective Population Sizes 

**Figure 6:**
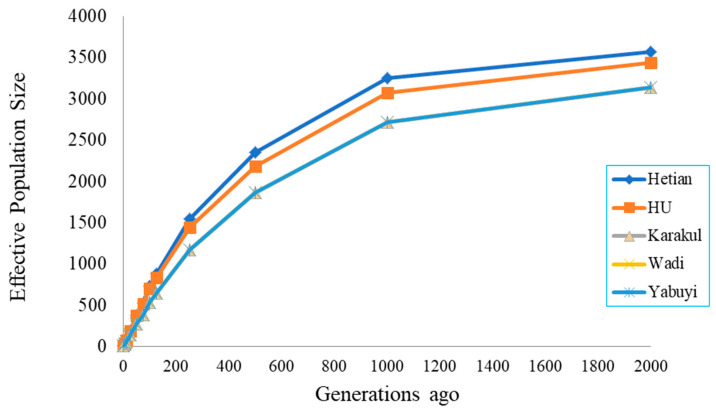
Estimated effective population sizes (Ne) over time for five Chinese native sheep breeds adapted to extremely dry and humid environments.

Ancient and modern effective population sizes (Ne) were obtained in these populations. We observed a rapid increase in N_e_ over the past 1000 generations and then a slow increase up to 2000 generations ago (Figure 6). Its decay over time indicated that the ancestral population based on 2000 past generations had a much larger N_e_ displayed for Hetian (*n* = 3563) and Hu (*n* = 3432) animals, compared to the most current generations. The N_e_ for the last 2000 generations showed the same value (*n* = 3133) shared in Wadi, Yabuyi and Karakul populations, falling above the minimum value (*n* = 30) of individuals for any livestock species to ensure the viability and genetic improvement in breeding programs. 

## 4. Discussion

In previous studies, we revealed the genomic diversity, population structure and selection signature characteristics of five sheep populations in China [45]. Here, we extended our analysis using runs of homozygosity, LD and N_e_ as useful tools for detecting genomic relatedness, providing population assessment historical information over time and predicting underlying genome architecture [46]. In this study, we used 600,000 high-density bead chips data to study the genome-wide runs of homozygosity, linkage disequilibrium and effective population size of five Chinese sheep breeds adapted to extreme dry and humid environments. The measurement of linkage disequilibrium provides some allelic frequency dependence in a limited sample size [47]. Secondary allele frequency is important because Linkage disequilibrium is a function of allelic frequency independent of the measures used, and low MAF may correspond to larger differences in coupled allele frequencies, which may result in lower LD estimates measured by *r*^2^ or D’ [48]. Therefore, filtration via quality control standards and thresholds could affect the distribution and extent of LD [49] because there is an important correlation between high levels of LD and high proportions of SNPs with high MAF value. However, in this study, we found that the MAF values ranged from 0.266 to 0.270, with an average mean of 0.268. These results are consistent with the previously observed MAF of 0.26 in Bonsmara and Drakensberg cattle and lower than the 0.28 value observed in Angus and Holstein cattle [50]. Inbreeding coefficient measures the percentage of the increase in homozygous gene pairs relative to the average number of breeds [51]. In the current study, the inbreeding coefficient ranged between −0.004 and −0.04, which is lower than the previous report of F_IS_ = 0.04 for Brazilian Santa Inés sheep [52], as well as lower than the values reported in Chinese Merino sheep, Italian sheep and Spanish sheep breeds [36,53,54]. In addition, it was lower than 0.29 value found of various cattle breeds in South Africa [50]. The main reason is that sheep from dry and wet environments have not been strictly selected and the effective population scale is large [10]. When the number of homozygous loci observed is lower than expected, a negative inbreeding coefficient will appear, which indicates the heterogeneity of the population is greater than expected. This may be due to the compound characteristics of breeds [52].

### 4.1. Linkage Disequilibrium

The linkage disequilibrium (LD) decay analysis showed that the breeding histories of five Chinese sheep breeds were distinct. Wadi, Karakul and Yabuyi displayed the highest LD decay, while Hetian and Hu sheep had the lowest LD across all genetic distance intervals (Figure S2). In general, the mean *r*^2^ values decreased rapidly with increasing genome distance and were constant after 200–300 kb. The most rapid decrease in r^2^ was observed for the first five bins. The phenomenon may be caused by the mixing effects. This result is consistent with that reported by Gibbs et al. [55].

### 4.2. Effective Population Size 

Effective population size is one of the most important parameters in population genetic and conservation biology. Its transforms census size of the actual population into the size of the ideal population, showing the same loss rate of genetic variation, increases of inbreeding, mutation accumulation and determination of the accuracy of genome selection [56]. Our results show a rapid growth trend in N_e_ of the five research groups in the past 1000 generations, and then it slowly increased up to 2000 generations ago across the five studied populations [11]. Interestingly, Hetian and Hu sheep showed higher N_e_ estimates before 2000 generations. Wadi, Karakul and Yabuyi sheep breeds had lower N_e_ estimates in all generations due to population admixture within the breeds [57]. Therefore, it is beneficial to consider our results in historical demographic context of Chinese sheep population. This finding is in line with our previous studies of genomic diversity, population structure and selection signature, in which we observed that principal component and admixture analysis clustered population corresponding to their geographical distribution and breeding history [45]. In general, the increase of the estimated N_e_ value across five sheep breeds generations ago suggest that these animals can ensure viability within breeds in breeding programs [58]. In addition, maintaining sufficient N_e_ is very important for maintaining heterozygosity and heterosis of Chinese animal breeds [59]. 

### 4.3. ROHs

Estimation of ROH is useful tool for exploring genetic diversity, providing information about the history of population demographic assessment and predicting potential genome structure [6,16,60]. As presented by Purfield et al. [6], relatively shorter ROH are most likely to be associated with ancestral inheritance or potential bottlenecks, while long ROH are more likely associated with relatively recent inbreeding. Our results show that ROH is common in all breeds, and some ROH length categories can be used as indicators of blood relationship and breed population history; selection pressures and breeding management impacts on sheep genome may potentially leave an imprint on ROH length. 

The total length of ROH in Yabuyi, Karakul and Wadi sheep were higher, which indicated that their genetic diversity was low. In general, the average ROH of all breeds ranged from 11.56 to 26.29 Mb, which was consistent with the results obtained in Swiss sheep [61]. The distribution patterns of ROH in Yabuyi, Karakul and Wadi sheep are different. They show that the average value of ROH is higher (>1–5 Mb) in the longer species, which is the performance of population reduction and recent inbreeding [62]. Therefore, the accumulation of long ROH in the genome of those breed enables them to carry harmful homozygous mutations [21]. The highest F_ROH_ value is Yabuyi sheep, followed by Wadi and Karakul sheep, which indicates that the inbreeding rate of these sheep breeds is very high. This may be due to a small number of rams being widely used in herds breeding. Therefore, extensive mating among relatives may be the reasons for the high proportion of fixed alleles, resulting in the low genetic diversity in Yabuyi, Karakul and Wadi. Attention should be paid to prevent loss of sheep genetic resources. However, Hetian sheep have limited inbreeding, followed by Hu sheep breeds, which reflects the proper management of breeds, sufficient effective population size (Ne) and large number [63]. 

The maximum frequency of Yabuyi, Karakul and Wadi sheep in the longest ROH category reflect that recent inbreeding probably exists in these breeds. Generally, the high proportion of long ROH segments in the population indicated strong inbreeding in recent generations. In animal breeding, if there is a genetic relationship between individuals, it will inevitably result in increased homozygosity of selective region and stronger genome selection signals [64]. In addition, these results are supported by the authors of [65] who reported that the number of ROH per individuals in the non-selected Holstein population is significantly lower than that of the two heavily selected populations in the United States. 

The distribution of ROH on each chromosome has a certain regularity. The first three chromosomes have the largest number of ROH, while the number of ROH gradually reduce with the decrease of chromosome length. The number of ROH on chromosome OAR20 is the lowest, with 13,838 segments. Our results are consistent with those reported in sheep [66] and goats [7]. The coverage of each chromosome is greatly different among different populations, suggesting that it may be species specific.

### 4.4. ROH Candidate Genes 

To identify genomic regions with the highest frequency of ROH and thus harboring candidate genes potentially under selection, we examined ROH genomic regions with inbreeding coefficients that span a variety of candidate genes directly or indirectly associated with adaptation to extremely dry and humid environments. Genes associated with innate and adaptive immune responses include humid and thermo-tolerance, disease resistance, energy and digestive metabolism and performance in extreme dry and humid environments. 

#### 4.4.1. Genes Related to Adaptive and Innate Immune Response

Several overlapping genes related to innate and adaptive immunity in mammals, such as *HERC2* and *CYFIP1*, were identified in this study, which are reported to regulate innate and acquired immune response, as well as cytokine signaling. In addition, *HERC2* and *CYFIP1*, which are involved in hemostasis regulation and mucosal defense, are also detected, which are of great significance in protecting sheep from parasites [67]. Furthermore, *HERC2* is a marker gene reported for biological predictions, some of which are unique and need functional verification [68].

#### 4.4.2. Genes Associated with Disease Resistance

Multiples candidate genes have been found in the genomic regions involved in host defense mechanism, diseases resistance and inflammation. The most significant genes include *5S_rRNA*, which is a gene detected in several overlapped regions (OAR2 and OAR6) with high statistical significance under warmer and humid climates conditions. It causes changes in the distribution and abundance of the five most common ticks in livestock in northern Xinjiang region of China. These ticks can transmit Borrelia disease in domestic animals [69]; control Lyme disease caused by *Borrelia* (*Burgdorferi sensu lato* complex is an important endemic zoonosis) in human, sheep and cattle [70]; and control Mycoplasma in sheep [71]. 

#### 4.4.3. Genes Associated with Body Weight and Digestive Metabolism Traits

Growth and body weight are the most important economic traits of livestock specially used for meat production. Several breeds from dry area or tropics tend to have small body weight/size and growth rate compared to humid or temperate breeds [72]. Bodyweight is one of the important characteristics of meat-type animals, which can be measured at birth or other life stages. Therefore, natural and artificial selection may leave genetic traces in the genome region of Chinese sheep related to wet and dry environments. We found overlapped candidate genes on the OAR10 chromosome, such as *GJB2* and *GJA3*, which are related to body size and developments [29]. Another gene, Centrosomal P4.1-associated protein (*CPAP*, also named *CENPJ*), is a centrosomal protein that participates in the assembly of centrioles and is very important for centrosome functions such as mitosis, motility or intracellular communication [73].

## 5. Conclusions 

In this paper, we highlight the homozygosity patterns, population inbreeding levels and effective population size of five diverse Chinese sheep breeds from dry and humid environments. Based on the study of the occurrence and distribution characteristics of ROH, different numbers and lengths of ROH were found. Through ROH analysis, we revealed different genomic regions and candidate genes, which are highly correlated with important traits. Yabuyi, Karakul and Wadi sheep had higher homozygosity, whereas the inbreeding of Hetian sheep was low, followed by Hu sheep, which shows that the breeding management is proper, and the effective population size is sufficient. Our work contributes to a better understanding of genetic variation among different domestic sheep breeds and provides new insights into the genetic inbreeding aspects of selection and population demographic events. 

## Figures and Tables

**Table 1:** Description of five native Chinese fat-tailed sheep breeds and their agro-ecological locations.

Breeds	Hetian (HT)	Karakul (KK)	Yabuyi (YY)	Wadi (WD)	Hu (HU)
Coat Color	White with pigmentation	Mainly black or grey	White	white with Pigmentation	White with Pigmentation
Body size	Medium	Large	Medium	Medium	Medium
Tail types	Short fat-tail	Long fat-tail	short fat-tail	Short fat-tail	Short fat-tail
Uses	Meat/Carpet wool	Lamb fur/meat	Meat	Meat/fur	Meat/lamb fur
Agro-ecology	Arid, lowland	Arid, semi-desert, Lowland	Arid, low land	Subhumid, lowland	Moist lowland
Community	Xinjiang/Hetian	Xinjiang	Xinjiang	Shandong/Binzhou	Jiangsu /Xuzhou
Temperature (°C)	−28.9 to 43.2	−20 to 33	−26 to 43	−16 to 39	−9.9 to 38.3
Average rainfall (mm)	150	150	35	592	930

**Table 2:** Summary of samples, inbreeding coefficients and minor allele’s frequency in five Chinese sheep breeds.

Breed Name	Acronym	Location	Sample Size (*n*)	Ecology	*F*_IS_	MAF < 0.05
Hetian	HT	Xinjiang	19	Arid land	−0.02	0.269
Karakul	KK	Xinjiang	20	Arid/Desert	−0.04	0.267
Yabuyi	YY	Xinjiang	20	Arid	−0.027	0.266
Wadi	WD	Shandong	17	Sub-Humid	−0.004	0.270
Hu	HU	Jiangsu	20	Humid	−0.03	0.268

Note: *F*_IS_, inbreeding coefficient; MAF, minor allele frequency.

**Table 3:** Summary of ROH segments proportions at different length categories across five Chinese breeds.

Length Category (Mb)	Hetian	Karakul	Yabuyi	Wadi	Hu
F_ROH_ (1–5)	28.14	73.27	84.82	81.53	49.66
F_ROH_ (5–10)	9.24	14.45	14.15	12.28	9.64
F_ROH_ (10–15)	7.05	8.24	3.06	2.59	1.86
F_ROH_ ≥ 15	1.81	0	3.12	1.03	1.21
Average	11.56	23.99	26.29	24.35	15.59

**Table 4:** The candidate genes located in genomic regions with the highest ROH frequency associated with important traits.

CHR	Positions	Gene Name	Functions	Gene Description
10	36,271,774–36,272,454	*GJB2*	Body size/development	gap junction protein beta 2
10	36,304,573–36,305,769	*GJA3*	Body size/development	gap junction protein alpha 3
10	36,669,829–36,713,175	*CENPJ*	Protein implements	centromere protein J
2	11,175,0152–11,175,0269	*5S_Rrna*	Disease resistance	5S ribosomal RNA
2	11,247,8388–11,272,0769	*HERC2*	Innate immune response	HECT and RLD domain containing E3 ubiquitin protein ligase 2
2	11,283,9853–11,294,4326	*CYFIP1*	Innate immune response	cytoplasmic FMR1 interacting protein1
6	79,425,129–79,425,234	*5S_rRNA*	Disease resistance	5S ribosomal RNA

## Supplementary Materials

The following are available online at https://www.mdpi.com/2073-4425/11/12/1480/s1, Figure S1. A correlation between the numbers of ROHs (n ROH) and the sum of all ROH segments; Figure S2. Linkage disequilibrium (LD) decay by distance across the five Chinese sheep breeds Hetian, Hu, Karakul, Wadi and Yabuyi. Table S1. Autozygosity islands were evident across the genome; Table S2. Go Terms with High ROH frequencies.

## Data Accessibility

The datasets analyzed in this study are available upon request from corresponding authors.
