# Supplementary material for: Genome-Wide Analysis Revealed Homozygosity and Demographic History of Five Chinese Sheep Breeds Adapted to Different Environments
Source: Genes (Basel). 2020 Dec 9;11(12):1480. doi: 10.3390/genes11121480 (PMC7764688; doi:10.3390/genes11121480)
Supplement: Supplementary file 1 [file genes-11-01480-s001.zip › Figuer S2.pdf]

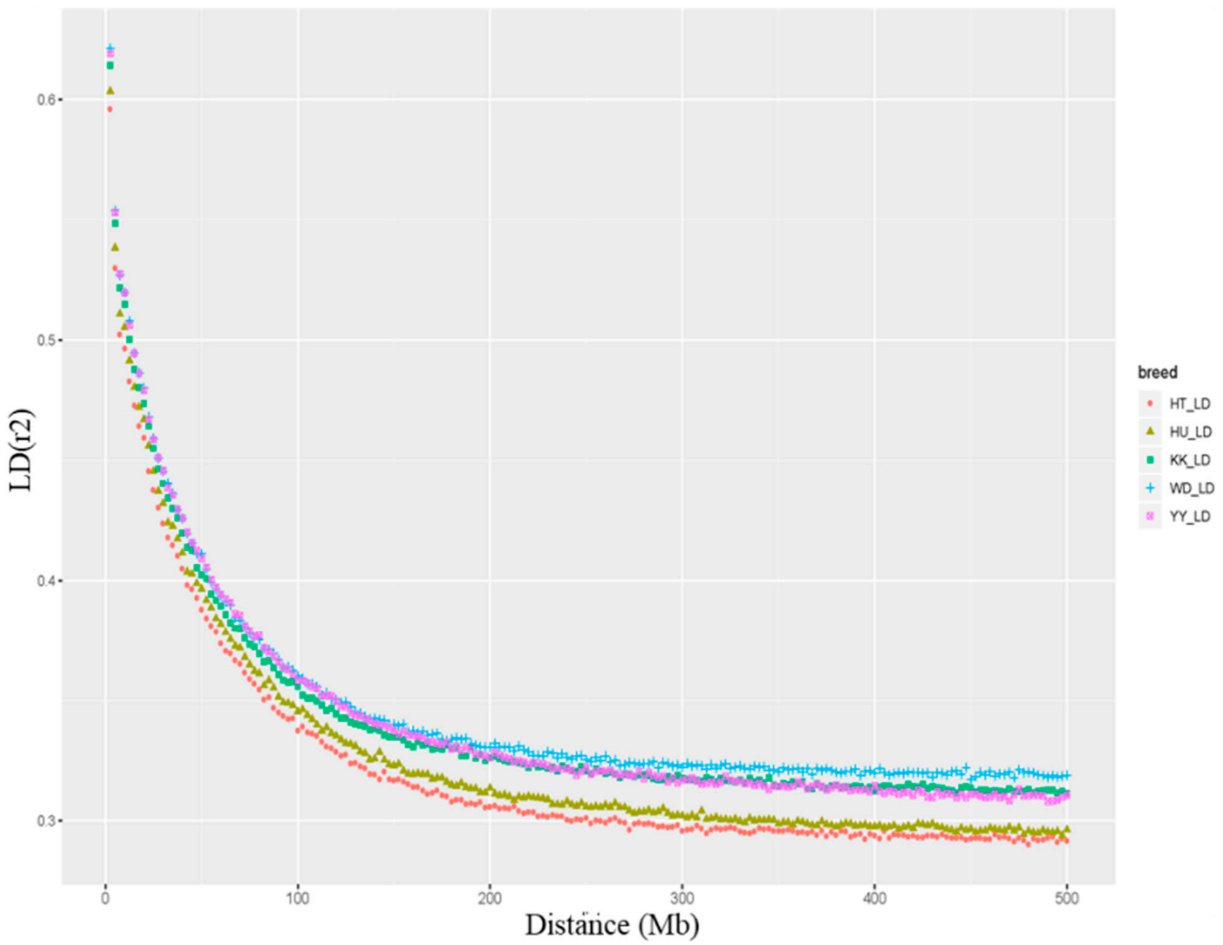

Figure S2. Linkage disequilibrium (LD) decay by distance across the five Chinese sheep breeds, Hetian (HT), Hu (HU), Karakul (KK), Wadi (WD) and Yabuyi (YY).
