# Supplementary figures and images for: Genome-Wide Analysis Revealed Homozygosity and Demographic History of Five Chinese Sheep Breeds Adapted to Different Environments
Source: Genes (Basel). 2020 Dec 9;11(12):1480. doi: 10.3390/genes11121480 (PMC7764688; doi:10.3390/genes11121480)

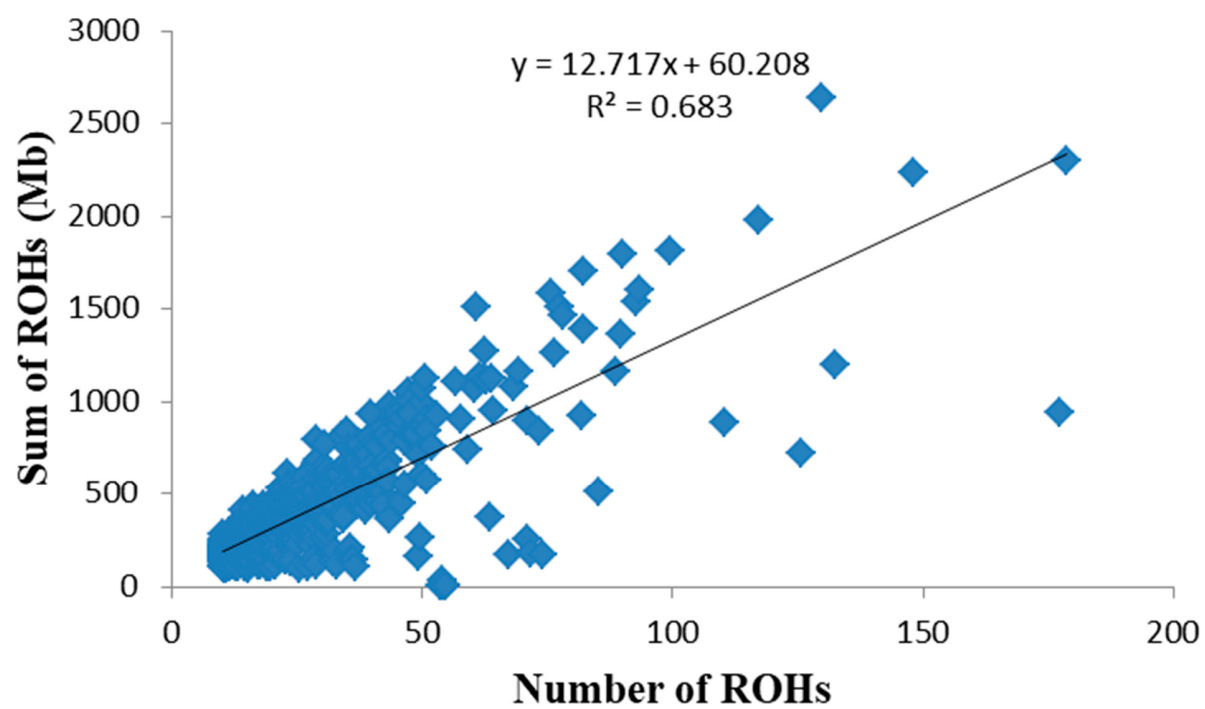

**Figure S1:** A correlation between the numbers of ROHs (n ROH) and the sum of all ROH segments.

Supplement: Supplementary file 1 [file genes-11-01480-s001.zip › Figuer S1.pdf]
